# Primary Retroperitoneal Serous Cyst Adenoma: A Case Report and Literature Review

**DOI:** 10.7759/cureus.45379

**Published:** 2023-09-16

**Authors:** Shariful Islam, Aneela Shah, Vijay Naraynsingh, Patrick Harnarayan

**Affiliations:** 1 Department of Surgery, San Fernando General Hospital, San Fernando, TTO; 2 Department of Clinical Surgical Science, University of the West Indies, Saint Augustine, TTO; 3 Department of Clinical Surgical Sciences, University of the West Indies, Saint Augustine, TTO; 4 Department of Surgery, Medical Associates Hospital, Saint Joseph, TTO; 5 Department of Surgery, San Fernado General Hospital, San Fernando, TTO

**Keywords:** massive mucinous cystadenoma, mucinous cystadenocarcinoma, retroperitoneal mass, serous cystadenomas, primary

## Abstract

Primary retroperitoneal serous cyst adenomas (PRSCs) are extremely rare thin-walled cystic lesions whose pathogenesis is not well understood. Clinical presentation varies depending on the lesion's size and location, i.e., larger lesions compress adjacent organs, giving the impression of malignancy. Although advances in imaging techniques enable to identify various characteristics of retroperitoneal cystic lesions, there are no pathognomonic signs to confirm the diagnosis. The exact diagnosis is based on the histology after complete surgical excision. An open surgical approach is considered the traditional method of complete resection; however, laparoscopic techniques have increasingly been employed. Diagnostic aspiration is discouraged due to the potential risk of seeding if the lesion is malignant. We present the case of a 51-year-old woman who underwent complete excision of a large right retroperitoneal cyst, histologically confirmed as PRSC with a review of the background and management options of this phenomenon.

## Introduction

Primary retroperitoneal serous cyst adenomas (PRSCs) arise from any location within the retroperitoneum. Their occurrence is extremely unusual because epithelial cells generally do not exist in this region. The exact pathogenesis of PRSC is not well understood. However, other lesions that can originate here include soft tissue sarcoma, malignant retroperitoneal cysts, retroperitoneal fibrosis, and lymphoma [1].

While radiology provides some information regarding the likely origin and features, the definitive diagnosis of PRSC is determined pathologically. Therefore, complete surgical resection is necessary once a cystic retroperitoneal lesion is identified. Yet, the optimal surgical approach remains to be determined [2,3].

We present a case of a 51-year-old woman with symptomatic uterine fibroids and chronic right upper quadrant pain. Imaging findings suggested a large mesenteric versus retroperitoneal cyst, and she underwent surgical resection. After a fairly uneventful postoperative course, final histology confirmed a completely excised retroperitoneal serous cystadenoma. This case report reviews the history and management of retroperitoneal serous cystadenomas.

## Case presentation

A 51-year-old female was referred to the surgical outpatient clinic with a one-year history of right-sided colicky abdominal pain and dysmenorrhea. She had no other associated gastrointestinal or gynecological symptoms. Her history included previous ultrasound-guided aspiration of a right retroperitoneal cyst four years prior, with lymphocytes and monocytes on cytology. She also had large uterine fibroids, with two previous uneventful myomectomies. She was a non-smoker and denied any other significant comorbid condition. 

On examination, her vital signs were within normal limits and abdominal examination revealed a lower midline scar and a palpable supra-pubic mass, likely uterus, just inferior to the umbilicus. 

The patient presented with abdominal ultrasound findings of a 10×8×4 cm simple intra-abdominal cyst just inferior to the gallbladder and lateral to the right kidney. It was reported to have an enlarged uterus with multiple large fibroids.

Blood investigations including complete blood count, kidney and liver function test, as well as serum tumor markers (CA 19-9, CEA, CA125) were within normal limits.

**Figure 1:**
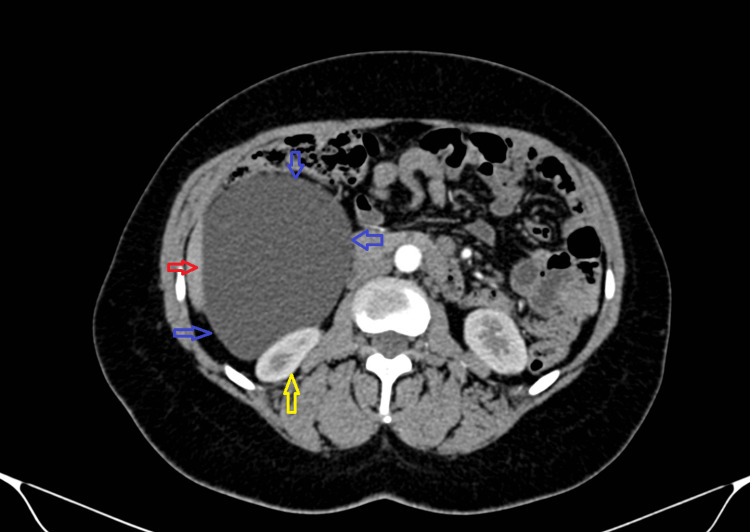
Axial CT image of abdomen and pelvis with intravenous contrast showing a (14x10x6cm) right retroperitoneal simple cyst (red arrow: liver; blue arrows: retro-peritoneal cyst; yellow arrow: right kidney) CT, computed tomography

**Figure 2:**
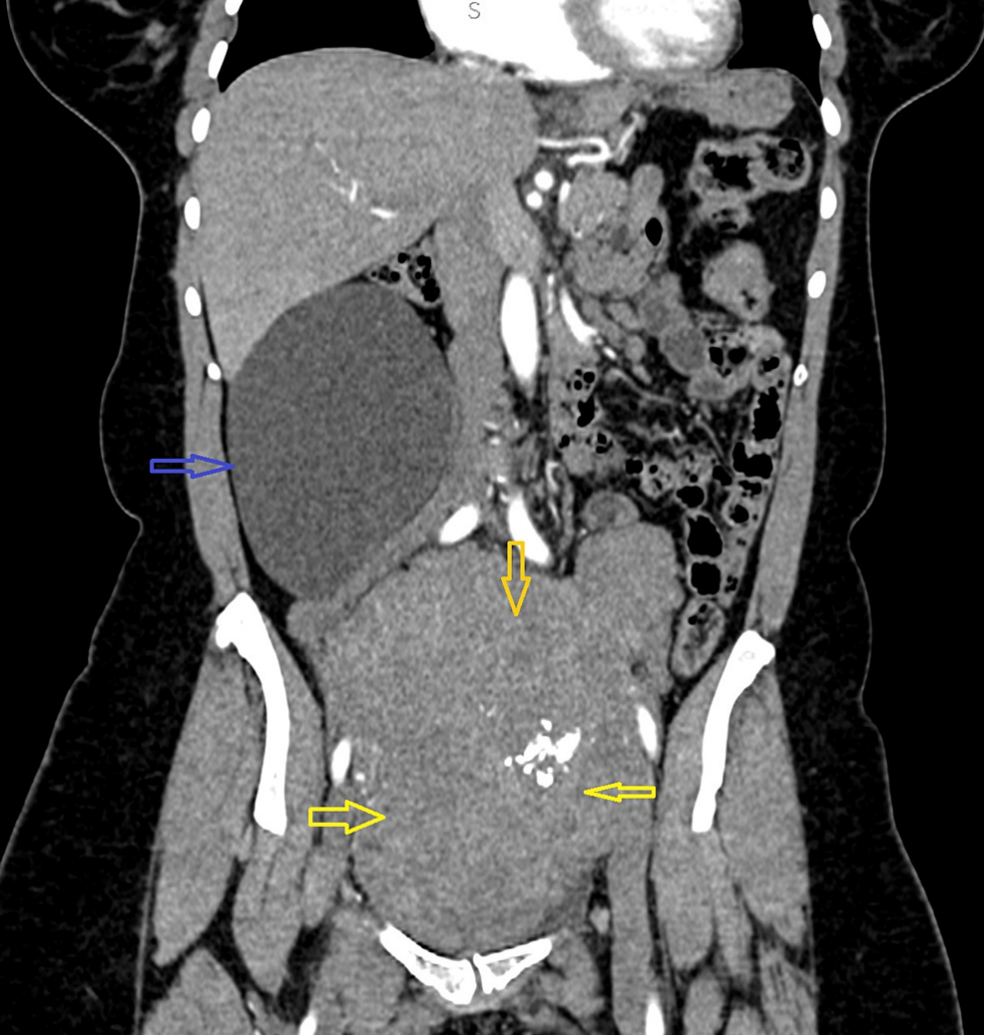
Coronal CT image of abdomen and pelvis with intravenous contrast showing the right-sided cystic lesion (blue arrow) and a large fibroid uterus (yellow arrows) CT, computed tomography

A computed tomography (CT) of the abdomen and pelvis with intravenous contrast was then performed, and it revealed a 14×10×6 cm retroperitoneal simple cyst inferior to the right lobe of the liver and lateral to the right kidney (Figure 1). It also noted the presence of a large fibroid uterus 16×10×10 cm (Figure 2).

**Figure 3:**
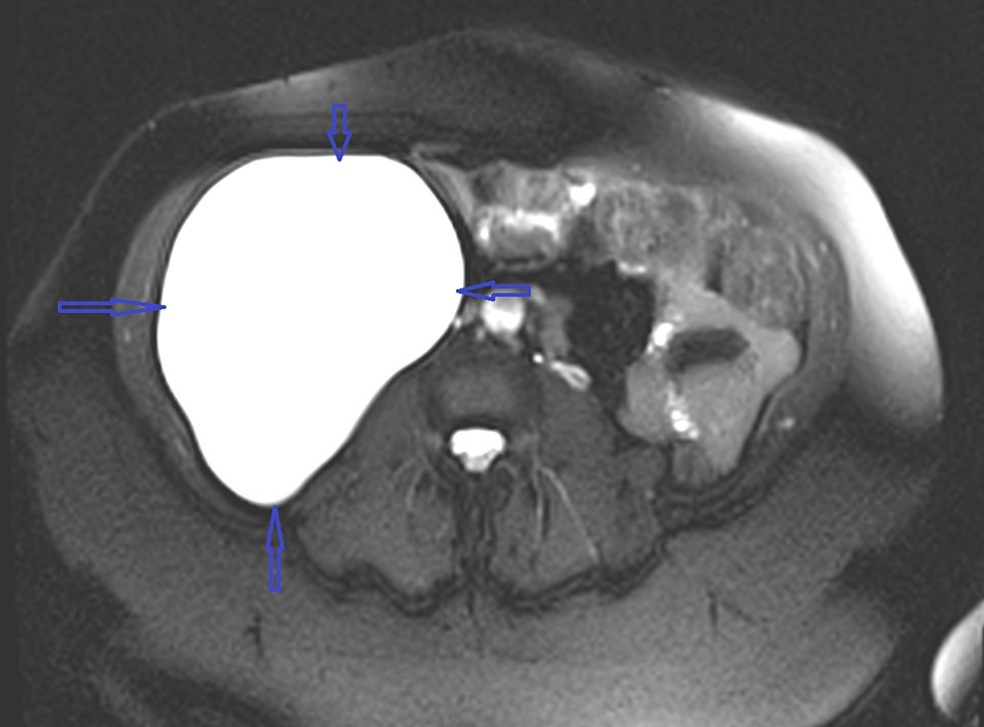
T-weighted MRI of abdomen image showing a 15x9x5 cm right retroperitoneal cystic mass MRI, magnetic resonance imaging

A magnetic resonance imaging (MRI) of the abdomen and pelvis revealed a uni-loculated intra-abdominal cystic mass (15×9×5 cm) inferior to the right lobe of the liver and inferior-lateral and inferior to the right kidney with resultant mass effect on the urinary bladder (Figure 3). A multi-fibroid uterus was also confirmed. The patient was referred to the gynecologist and the decision was made to perform both procedures at the same time.

**Figure 4:**
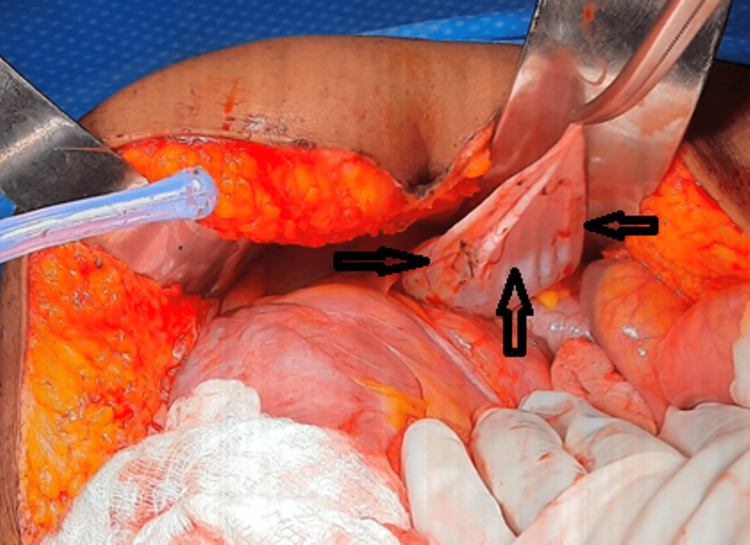
Intraoperative image showing the large right retroperitoneal cyst (black arrows)

The patient consented to joint excision of the mesenteric cyst and total abdominal hysterectomy with bilateral scalping-oophorectomy. Under gestational age with endotracheal intubation, bilateral ureteric stenting was performed. A midline laparotomy and adhesiolysis were performed; a right retroperitoneal cystic lesion was identified, and there was no other mesenteric lesion. Hysterectomy with bilateral salpingo-oophorectomy was performed first to remove the bulky uterus and to improve access to the retroperitoneal cyst. The right colon was then mobilized medially, allowing complete excision of the smooth-walled cystic mass, without rupture (Figure 4).

**Figure 5:**
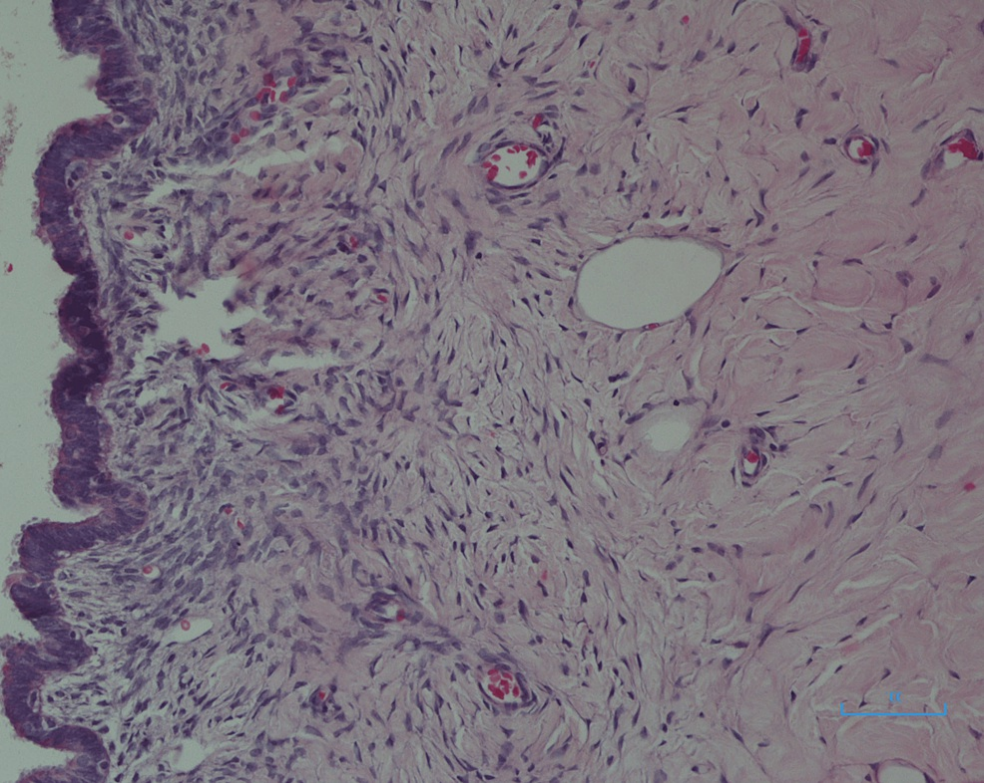
Serous cyst adenoma: histo-micrograph showing cyst wall lined by epithelium with abundant eosinophilic cytoplasm (hematoxylin and eosin stain)

The postoperative recovery of the patient was uneventful and discharged home on day four. The final histopathology report revealed that it was a retroperitoneal serous cyst adenoma (Figure 5). The case was discussed at our multi-disciplinary team meeting, and the decision was made for routine surveillance to monitor any future recurrence.

The patient was followed up in the surgical outpatient clinic and at 18 months of follow-up, the patient was doing well with no further complaints. A repeat CT scan of the abdomen at one year revealed no recurrence of the disease. 

## Discussion

Retroperitoneal serous cystadenoma was first described by Staehlin in 1915 [4]. It is a cystic lesion derived primarily from the retroperitoneal space, with no direct communication with other viscera. Unlike secondary lesions, primary retroperitoneal cystic lesions are rare because the retroperitoneum generally lacks epithelial cells. As such, the incidence of PRSCs is difficult to estimate [1,5]. However, these patients have ranged in age from 28 to 79 years and are exclusively female based on the reported cases [1,6,7]. The pathogenesis of PRSCs is also not well understood. Different hypotheses have been described in the literature and include remnants of embryonal urogenital apparatus, heterotopic ovarian tissue, and enteric duplication cysts [1,5]. 

The symptomatology of PRSCs typically depends on their size and location. Most patients remain asymptomatic for a long time because the large retroperitoneal space accommodates cyst growth [7]. When symptomatic they commonly present as a palpable abdominal mass or, as in this case, with abdominal pain. Other symptoms like fever, decreased appetite, and or weight loss may indicate underlying malignancy [1,6].

It is important to note that there are no pathognomonic signs or symptoms of PRSCs. The same is also true of laboratory and radiological features. Few case reports suggest an association with elevated serum tumor markers like CEA, CA125, CA19-9, and CA15-3. Some reports also suggest a diagnostic value of tumor markers in aspirated cystic fluid, but these are neither sensitive nor specific [1,5-8].

In terms of imaging, ultrasound of the abdomen lacks specificity [9]. Both CT and MRI can be used for the diagnosis of this condition; however, none of the modalities can exclude the malignant potentials of PRSCs [10-12].

Despite this fact, MRI is considered a better modality than CT when assessing retroperitoneal lesions. MRI allows better assessment for internal septae and mural nodules, local extent, and likely organ of origin, if not primarily retroperitoneal [1,6,13].

Differentiating PRSCs from other potentially malignant cystic tumors is crucial. Unfortunately, however, this can only be distinguished pathologically (once the lesion is excised surgically) [14]. The classic macroscopic description of a PRSC, as shown in Figure 5, is a smooth, unilocular cyst, encapsulated by a thin layer of fibrous tissue in the retroperitoneal space and no direct involvement with intra-abdominal viscera [6].

Diagnostic cyst aspiration, as happened in this case before she was referred, may improve symptoms but is not generally recommended due to concerns of potential tumor seeding if the lesion is indeed malignant. Whether PRSCs have malignant potential is yet to be determined as previous reports do not indicate this progressive pattern [7].

The ideal treatment for PRSCs is complete surgical excision: the surgical approach depends on the location and size of the lesion, as well as the expertise of the surgeon [1].

Historically, open enucleation was the recommended approach. However, laparoscopic (transabdominal or retroperitoneal) excision has become more popular. The transabdominal approach enables a large working space but requires mobilization of the colon for access [13,15]. The retroperitoneal approach has a smaller working space, but if the cystic contents spill, there is a smaller risk of abdominal dissemination [7].

On the other hand, some reports suggest that laparoscopic excision should be avoided when the pathology of the cystic lesion remains unknown or suspicious of malignancy. In this case, the patient’s enlarged fibroid uterus precluded the intraoperative advantages a laparoscopic approach may have provided and an open approach seemed to be more feasible.

So far, no recurrences have been reported following surgical treatment of PRSCs, unlike an estimated 25% recurrence rate for malignant retroperitoneal cystic lesions [1,7]. However, because of the unknown risk of recurrence of this entity, the question of whether surveillance post-excision is needed remains unanswered.

## Conclusions

PRSCs are extremely rare and often remain asymptomatic until it is very large. Although CT and MRI scans enable us to identify various characteristics of retroperitoneal cystic lesions, the exact diagnosis is based on histology after complete surgical excision. Diagnostic aspiration is discouraged due to the potential risk of seeding if the lesion is malignant.
